# Encapsulation of Disease-Causing and Commensal Mitis Group Non-Pneumococcal Streptococci

**DOI:** 10.3390/pathogens14090876

**Published:** 2025-09-02

**Authors:** Daniel M. Musher, Mathias Müsken, M. John Hicks, Lesley McGee, Bernard Beall

**Affiliations:** 1 Michael E. DeBakey Veterans Affairs Medical Center, Houston, TX 77030, USA; 2 Baylor College of Medicine, Houston, TX 77030, USA; mjhicks@texaschildrenshospital.org; 3 Helmholtz Centre for Infection Research, 38124 Braunschweig, Germany; mathias.muesken@helmholtz-hzi.de; 4 Texas Children’s Hospital, Houston, TX 77030, USA; 5 Centers for Disease Control and Prevention, Atlanta, GA 30333, USA; lmcgee@cdc.gov (L.M.); beb0@cdc.gov (B.B.)

**Keywords:** *Streptococcus pneumoniae*, *Streptococcus mitis*, viridans, streptococci, community acquired pneumonia, commensal bacteria, capsules

## Abstract

**Background**: Mitis group non-pneumococcal streptococci (MGNPS), specifically *Streptococcus mitis*, *Streptococcus infantis* and *Streptococcus oralis*, have recently been shown to cause pneumonia and/or bacteremia. These organisms often have capsular (*cps*) operons resembling those in pneumococci, and some express *cps*-generated polysaccharides that antigenically cross-react with pneumococcal capsular serotypes. But, to date, a series of MGNPS isolates has not been studied by electron microscopy (EM) for the presence of a capsule. **Methods**: We studied 21 MGNPS; 11 were isolated from sputum and determined to have caused pneumonia, 3 were isolated from blood, and 7 were commensal isolates cultured from the oral cavity of healthy adults. Two reacted with a pneumococcal anticapsular antibody. Isolates were fixed with two different protocols and examined by transmission EM. **Results**: EM of MGNPS after standard fixation and staining with uranyl acetate did not show capsules. In contrast, the 21 MGNPS isolates that we studied after fixation with ruthenium red and lysine acetate were all shown to be encapsulated. The thickness and density of capsules was related to their species: *Streptococcus pneumoniae* had the most prominent encapsulation and *Streptococcus oralis* had the least. However, within a species, there was no apparent difference in capsules between disease-causing and colonizing strains. **Conclusions**: EM with ruthenium red staining demonstrated capsules on 21 MGNPS, but within a species, there was no apparent difference between disease-causing and commensal isolates. It seems reasonable to conclude that the capsule, together with inoculum size, host’s ability to clear aspirated organisms, and other as yet unidentified virulence factors, all contribute to the pathogenesis of MGNPS pneumonia.

## 1. Introduction

*Streptococcus pneumoniae* and the closely related *Streptococcus mitis* are thought to have evolved from a common precursor [1]. *S. pneumoniae* was one of the first bacteria to be identified as an important pathogen for humans. It was the first to be shown to have a polysaccharide capsule that played an important role in virulence and one to which antibody was protective [2,3]. Capsules were originally identified by the *Quellung* reaction [2] and later by immunodiffusion. In contrast, except for their long-recognized role in causing subacute bacterial endocarditis, mitis group non-pneumococcal streptococci (MGNPS) have generally not been regarded as important causes of human disease and are often grouped under the label ‘commensal organisms’ [4,5]. However, studies in the past three decades have shown that MGNPS, specifically *Streptococcus mitis*, *Streptococcus infantis*, and *Streptococcus oralis*, cause bacteremia in severely immunocompromised patients [6,7,8,9]. A recent study of community-acquired pneumonia in middle-aged persons who have usual age-associated comorbid conditions but are not regarded as immunocompromised implicated MGNPS as the cause in nearly 20% of cases [10].

It is unclear what factor (s) might render these so-called commensal streptococci [4,5] virulent. Immunological methods—*Quellung* reaction and immunodiffusion—have shown that occasional MGNPS strains express polysaccharides identifiable with pneumococcal typing sera [11,12,13,14,15,16], although most do not. Most strains of *S. mitis*, *S. oralis*, and *S. infantis* that have been examined carry full-length *cps* operons, some of which are highly similar to their pneumococcal counterparts [4,11,12,13,14], and some individual strains that react with antibodies to pneumococcal capsular polysaccharides have been shown, by electron microscopy, to have capsules [17,18]. There is ample evidence for serologic cross-reactivity among pneumococci and a minority of MGNPS [19]. To our knowledge, however, no study to date has simply examined a series of MGNPS isolates for the presence of capsule. Further, none has compared infecting to colonizing (‘commensal’) strains of for the presence of capsule. It was shown by Hammerschmidt and Rohde that the fixation of *Streptococcus pneumoniae* with lysine acetate, ruthenium red, and osmium (LRR fixation) preserved the highly hydrated structure of capsules, allowing serotype differentiation by the bound capsular material [20]. Therefore, the goal of the present study was to utilize electron microscopy with LRR fixation to determine whether these streptococcal species have demonstrable capsules, and if so, whether capsules are present in infecting strains, commensal (colonizing) strains, or both.

## 2. Methods

### 2.1. Organisms

Twenty-one MGNPS were assigned species employing core genome phylogeny as previously described by investigators at the Streptococcus Laboratory, Respiratory Diseases Branch, Division of Bacterial Diseases, Centers for Disease Control and Prevention (Atlanta, GA, USA) [15]; twelve were speciated as *S. mitis*, five as *S. infantis* and four as *S. oralis.* Fourteen of the twenty-one organisms were identified as the cause of infection. Of these, 11 were isolated from sputum and met the criteria for having caused community-acquired pneumonia [10], having been seen as the sole bacterium by microscopic examination of a Gram-stained specimen and quantitated to >10^6^ cfu/mL in purulent sputum from a patient with pneumonia with no other recognized bacterial pathogen. Three were obtained by blood culture: one from a patient hospitalized for fever and cough with a newly recognized pulmonary infiltrate and two from the blood culture of febrile neutropenic patients (one of these was kindly provided by Dr. Samuel Shelburne) [8]. The remaining 7 MGNPS were isolated by culture of the oral cavity of healthy adults. Whole-genome sequencing employed a previously described bioinformatics pipeline for identifying pneumococcal serotypes [21].

### 2.2. Electron Microscopy

Bacteria were cultured on sheep blood agar, and colonies were fixed in glutaraldehyde, embedded in plastic, and then sectioned with an ultramicrotome. Specimens were initially stained with uranyl acetate and lead citrate and examined using transmission electron microscopy. Additionally, specimens were re-examined by the LRR fixation method described by Hammerschmidt and Rohde [20], a method that more definitively demonstrates capsular glycocalyx [22]. The LRR method is a stepwise protocol using lysine acetate, ruthenium red, and osmium tetroxide to fix overnight cultures of streptococci grown in 14 mL THY medium (15 mL Falcons) without shaking at 37 °C. After fixation, plastic embedding and staining with uranyl acetate and lead citrate was performed, as mentioned. Digital images were reviewed for the presence of capsules using a Libra 120 transmission electron microscope (Zeiss, Oberkochen, Germany) run at 120 kV. *Streptococcus pneumoniae* serotypes 5 and 15C served as positive controls.

### 2.3. Research and Publication Ethics

Review of medical records of patients discharged with a diagnosis of community acquired pneumonia without consent forms is approved by the IRB at Baylor College of Medicine (Protocol H-19318, Community-Acquired Pneumonia Quality Assurance Registry), expiration date 19 April 2026. This research involved the use of discarded samples from the Clinical Microbiology Laboratory at the Michael E. DeBakey VA Medical Center; this work is approved by the IRB at Baylor College of Medicine under Protocol H-0006, Infectious Disease Research Laboratory.

## 3. Results

### 3.1. Molecular and Genetic Characterization

All MGNPS isolates studied had recognizable *cps* operons. Conserved *wzg*, *wzh*, *wzd*, and *wze cps* operon genes shared 50–80% sequence identity with pneumococcal counterparts, and all strains appeared to contain diverse non-conserved serotype-specific loci as seen in pneumococci. Two isolates were assigned a pneumococcal serotype or serogroup through the pneumococcal typing pipeline (one serogroup 35C/42 *S. infantis* disease isolate and one serotype 5 *S. mitis* carriage [commensal] strain) based upon serotype-specific gene sequences (*wzy35C* and *wzy5*, respectively). The serotype 5 strain was positive for the pneumococcal serotype 5 polysaccharide repeat unit polymerase gene *wzy5*, with 86.6% sequence identity to the identically sized (1206 bp) pneumococcal counterpart. This strain produced serotype 5 antigen that appeared to be identical to pneumococcal serotype 5 as shown by immunodiffusion, although at a lower level, and gave a positive *Quellung* result after reaction with pneumococcal serotype 5 antiserum. This strain was also unique among the 21 isolates in carrying a *ply* gene that shared 97% sequence identity with pneumococcal counterparts. Two of twelve disease-causing isolates and five of seven colonizing isolates carried *ply* alleles with low homology (58–60% identity) to pneumococcal *ply.*

### 3.2. Electron Microscopy

**Figure 1:**
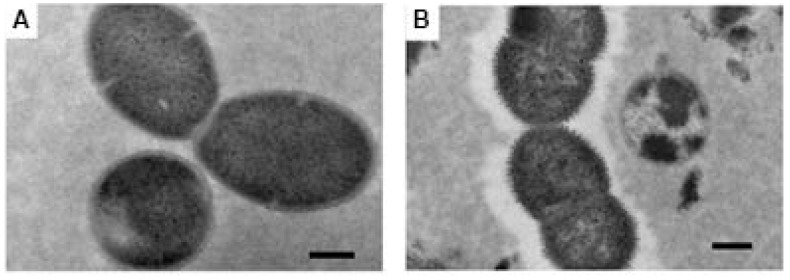
Transmission electron microscopy images after standard fixation protocol. (**A**) *Streptococcus mitis* showing no capsule; and (**B**) *S. pneumoniae* serotype 5, showing clear evidence of a capsule. Scale bar 200 nm.

Examination by transmission electronic microscopy following a standard fixation protocol and staining with uranyl acetate failed to identify recognizable capsule in any of the 14 isolates of *S. mitis*, *S. oralis*, or *S. infantis* that had been identified as etiologic agents of disease, or the 7 isolates obtained by oral culture from healthy adults (representative *S. mitis* shown in Figure 1A). Two strains of *Streptococcus pneumoniae* which served as positive controls were shown, by this technique, to have dense capsules, as seen in Figure 1B.

**Figure 2:**
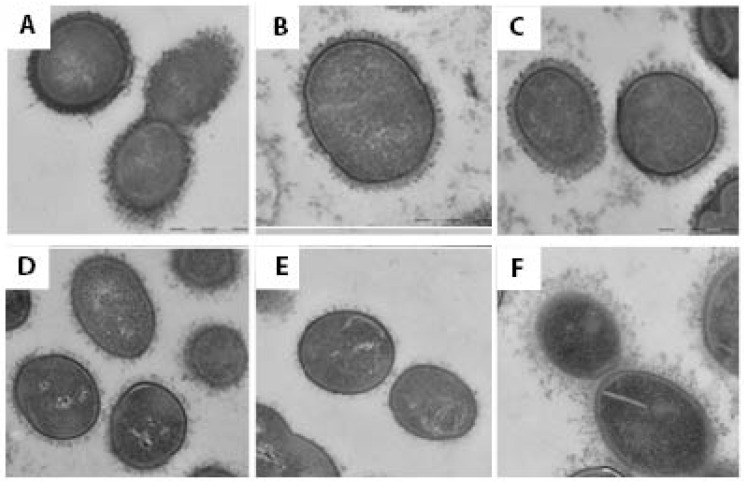
Transmission electron microscopy images after ruthenium red/lysine acetate fixation protocol. (**A**) *S. pneumoniae*, type 15C, sputum isolate, patient with pneumonia. (**B**) *S. mitis*, sputum isolate, patient with pneumonia. (**C**) *S. mitis* commensal isolate from the mouth of a healthy adult. (**D**) *S. oralis*, sputum isolate, patient with pneumonia; (**E**) *S. oralis*, commensal isolate from the mouth of a healthy adult. (**F**) *S. mitis*, commensal isolate with thick capsule, predicted as type 5 through the CDC bioformatics pipeline and cross reacted with antiserum to *S. pneumoniae* type 5. Scale bar 500 nm.

**Figure 3:**
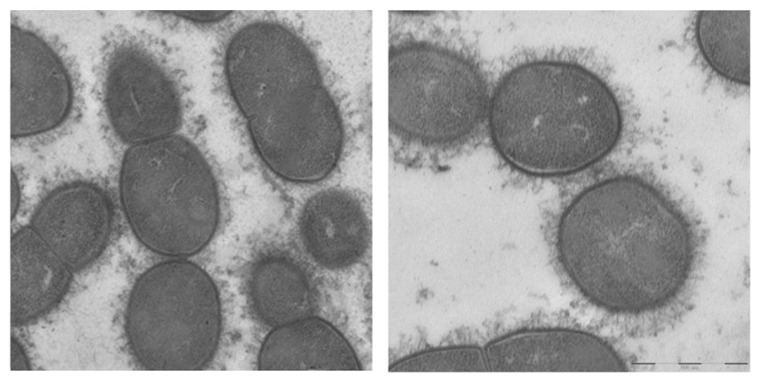
*Streptococcus infantis*, left panel. *Streptococcus infantis* that cross-reacts with antiserum to *S. pneumoniae* serotype 35C/42, right panel. Scale bar 500 nm.

In contrast, an electron microscopic study after a stepwise fixation protocol including ruthenium red and lysine acetate revealed capsular material in every MGNPS isolate examined (Figure 2). The capsule was most dense in *S. pneumoniae* (Figure 2A) and slightly less dense and more filamentous in *S. mitis* (Figure 2B,C) and *S. infantis* (not shown)*,* with scantiest capsule material in isolates of *S. oralis* (Figure 2D,E). Nonetheless, the *S. oralis* in Figure 2D caused pneumonia. One strain of *S. mitis* that contained sequences consistent with *S. pneumoniae* serotype 5 and was identified as serotype 5 through the CDC bioformatics pipeline had a capsule that was thick, although not as dense as that seen on pneumococci (Figure 2F). A strain of *S. infantis* that cross-reacted with an antibody to *S. pneumoniae* serotype 35C/42 was encapsulated as were other strains of *S. infantis* that did not do so (Figure 3). Importantly, within each MGNPS species, when colonizing (commensal) strains were compared to disease-causing strains, no apparent differences in the capsules were noted.

## 4. Discussion

Our recent study that implicated MGNPS as a cause of nearly 20% of cases of community-acquired pneumonia [10] motivated the present attempt to determine whether these organisms are encapsulated. Immunodiffusion and *Quellung* techniques have shown that a minority of MGNPS react with antiserum to pneumococcal capsular polysaccharides [22]; in a few instances, electron microscopy has shown these organisms to be encapsulated [17,18]. Most MGNPS examined to date have *cps* operons that might encode capsule production [4,12,13,14,22]. However, to our knowledge, no one has systematically examined a series of MGNPS for the presence of capsules by electron microscopy with appropriate fixation. A possible explanation why capsules of MGNPS have not been regularly described previously is that, as shown in Figure 1, transmission electron microscopy with standard glutaraldehyde fixation fails to show them since the capsular structures are likely destroyed due to glutaraldehyde [20]. In the present study, we therefore used the LRR fixation method, which has previously been shown to enable the preservation of the capsular structure of *Streptococcus pneumoniae* [20], as well as *Acinetobacter baumannii* [23]. Indeed, the method showed capsules on all commensal and infecting MGNPS that we studied, a finding that raises interesting questions about bacterial pathogenicity and host factors that contribute to pneumonia.

The thickness and density of capsular material appeared to be related to streptococcal species but not specifically to the commensal (colonizing) or disease-causing status of the organism. *S. mitis*, the species most closely related to *S. pneumoniae*, and *S. infantis* had capsules that were thick, although less dense than pneumococci, while capsules of *S. oralis* were thinner and less continuous. In one *S. mitis* strain that had a capsule consistent with *S. pneumoniae* serotype 5, the capsule was less dense but recognizably thicker than that seen in pneumococcus. Commensal isolates had capsules that were as prominent as those that caused pneumonia. It is worth noting that there remain important, albeit poorly understood, physicochemical differences between pneumococcal and MGNPS capsules, as shown in the present study by the differing abilities to detect the polysaccharide capsules with the two staining techniques and also as demonstrated by Marshall et al. using atomic force microscopy [18].

The presence of a capsule is the principal factor that enables *S. pneumoniae* to escape phagocytosis. Serotype-specific anticapsular antibody opsonizes pneumococci, thereby providing protection in experimental animals and in humans [24]. Some MGNPS express antigenically recognizable pneumococcal capsular serotypes, and immunization of experimental animals with these organisms stimulates an opsonizing antibody against pneumococci of the corresponding serotypes [11,14,17]. Accumulated data [12,13,14,15] indicate that the presence of pneumococcal serotype-specific genes within MGNPS correlates very well with reactivity with pneumococcal typing antisera. Our results are consistent with the concept that although most MGNPS are encapsulated, the majority do not express pneumococcal serotypes. That being said, the abundance, in some geographical regions, of MGNPS carrying pneumococcal serotype-specific genes, as assessed by PCR, can be surprisingly high [25].

Colonization by *S. pneumoniae* stimulates production of protective anticapsular antibody [26,27]. Studies in mice have shown that viridans streptococci that carry a pneumococcal capsule may stimulate a protective antibody against the relevant pneumococcal serotype [28]. Colonization of humans by a non-pneumococcal streptococcus that carries a type-specific capsule may stimulate opsonizing antibody to the relevant serotype pneumococcus.

Regarding other virulence factors, neumolysin, a conserved toxin common to all *S. pneumoniae*, plays a central role in pathogenesis of pneumococcal pneumonia. Intratracheal inoculation of pneumolysin into rodents causes typical pneumonia [29], and anti-pneumolysin antibody is protective [30]. Denapaite et al. [6] reported that 10 strains of *S. mitis* lacked the *ply* gene that encodes pneumolysin. Two of the twelve disease-causing MGNPS and 5 of 7 commensal isolates described in the present manuscript carried *ply* alleles, but with the exception of one colonizing strain of *S. mitis* that reacted with antiserum to *S. pneumoniae* serotype 5, these had low homology (58–60% identity) to pneumococcal *ply*. Genes with homology to pneumococcal choline-binding protein genes were readily screened from strains of all three species. However, homology to pneumococcal hyaluronidase and pilin backbone genes was absent. A limitation of the present report is the relatively small number of MGNPS studied, but the principle of encapsulation of MGNPS seems to be well established by the uniformity of the electron microscopic findings in all 21 strains we studied.

The lack of difference in capsule between commensal and infecting strains is consistent with the recent report by Kalizang’oma [31] that showed high genetic diversity amongst *S. mitis* strains but no pattern that was consistent with virulence. If the presence of a capsule or other recognized virulence factors does not clearly distinguish disease-causing from colonizing isolates, what might explain the pathogenicity of MGNPS? All of our patients who had pneumonia caused by these so-called commensal organisms had at least two conditions that predispose to bacterial pneumonia [10], but so did 95% of older male patients who were hospitalized with pneumonia due to *S. pneumoniae* [32], although it is difficult to compare the severity of these underlying conditions. A possible explanation for the occurrence of MGNPS pneumonia in some persons but not others is the inoculum size during microaspiration of oropharyngeal contents, which precedes and leads to pneumonia, but this is simply unmeasurable. In mice, the inoculum size of aspirated pneumococci is a major determinant of whether disease results [33]. It is likely that this same principle applies to other infecting bacteria in humans. Aspiration of a sufficiently large inoculum of bacteria with relatively low virulence may cause bacterial pneumonia in a human host who has poor glottal function, diminished clearance due to disrupted ciliary function because of chronic inflammation or viral coinfection, or poorly functioning phagocytic cells. Previous investigators have questioned the usage of the term ‘commensal,’ especially as it applies to MGNPS [4,5]. The relevance of this question is reinforced by the findings of the present study, and the demonstration of capsules in MGNPS may help to explain their role in causing pneumonia.

In conclusion, our findings, albeit based on a small sample, suggest that many MGNPS, whether colonizing or disease-causing, are encapsulated. *Streptococcus mitis*, the species most closely related to pneumococcus has the most clearly defined capsules, but other MGNPS have capsules as well. We did not, however, find a difference in the thickness or density of the capsule when we compared colonizing with disease-causing isolates. We propose that when MGNPS cause pneumonia, microaspiration is responsible, and as is the case with other bacteria, especially *Streptococcus pneumoniae,* the outcome is largely dependent upon the magnitude of the bacterial inoculum and the ability of the host to clear it. It seems reasonable to regard the capsule as contributing to virulence, although the precise role of the capsule in MGNPS remains to be elucidated.

## Data Availability

The original contributions presented in this study are included in the article. Further inquiries can be directed to the corresponding author.
